# Glutathione-linked enzymes in benign and malignant oesophageal tissue

**DOI:** 10.1038/sj.bjc.6690317

**Published:** 1999-04

**Authors:** R D Levy, M M J Oosthuizen, E Degiannis, D Greyling, C Hatzitheofilou

**Affiliations:** Masonic Research Fund [MRF] Biochemistry Laboratory, Department of Surgery, University of the Witwatersrand Medical School, 7 York Road, Parktown, 2193 Johannesburg, South Africa

**Keywords:** GSH-peroxidase, GSSG-reductase, GSH-s-transferase P1-isoenzyme, oesophageal cancer

## Abstract

Oxyradicals are involved in multiple mutational events and can contribute to the conversion of healthy cells to cancer cells. Glutathione (GSH) and the GSH-replenishing enzymes keep the antioxidant status of normal cells at a level where they can avert oxyradical derived mutations. The aim of this study was to determine whether in cancer cells the GSH-replenishing, GSH antioxidant and GSH-depleting enzymes were not at appropriate levels and therefore not able to protect cancer cells adequately against oxyradical-induced mutations. Cancer of the oesophagus was chosen since it is the most common gastrointestinal malignancy in South African Blacks. Biopsies and blood from 31 patients with cancer of the oesophagus and 29 non-cancer patients were assessed for these enzymes. The mean activity of the antioxidant and depleting enzyme GSH-peroxidase was elevated significantly by twofold in the cancer tissue compared to normal tissue. However, the activity of the replenishing enzyme GSSG-reductase and the level of the depleting enzyme GSH-s-transferase P1-isoenzyme were significantly reduced by 23% and 33% respectively. As in a previous paper we found that GSH was depleted and γ-glutamine transpeptidase was diminished in oesophageal cancer. There can be two reasons for GSH depletion. Firstly, elevated GSH–peroxidase will use more GSH in an attempt to cope with the excessive production of oxyradicals as revealed by elevated lipid peroxidation; this was, as shown by us before, elevated sixfold in oesophageal cancer. Secondly, if little replenishment of GSH occurred the level of GSH would become lower. This was confirmed by our findings that the activities of the replenishing enzymes were significantly diminished in oesophageal cancer tissue. Contrary to what was expected, the other depleting enzyme GSH-s-transferase P1 was not elevated in cancer tissue but was significantly lower. However, in the blood of the same patients it was significantly elevated. An explanation for this phenomenon is that, although the production of GST-P1 was enhanced in cancer, it did not show because it was rapidly extruded into the blood by an unknown mechanism operational only in cancer cells. © 1999 Cancer Research Campaign


*British Journal of Cancer* (1999) **80**(1/2), 32–37
© 1999 Cancer Research Campaign
Article no. bjoc.1998.0317

### Glutathione-linked enzymes in benign and malignant oesophageal tissue

**RD Levy, MMJ Oosthuizen, E Degiannis, D Greyling and C Hatzitheofilou**

Masonic Research Fund [MRF] Biochemistry Laboratory, Department of Surgery, University of the Witwatersrand Medical School, 7 York Road, Parktown, 2193 Johannesburg, South Africa

**Summary** Oxyradicals are involved in multiple mutational events and can contribute to the conversion of healthy cells to cancer cells. Glutathione (GSH) and the GSH-replenishing enzymes keep the antioxidant status of normal cells at a level where they can avert oxyradical derived mutations. The aim of this study was to determine whether in cancer cells the GSH-replenishing, GSH antioxidant and GSH-depleting enzymes were not at appropriate levels and therefore not able to protect cancer cells adequately against oxyradical-induced mutations. Cancer of the oesophagus was chosen since it is the most common gastrointestinal malignancy in South African Blacks. Biopsies and blood from 31 patients with cancer of the oesophagus and 29 non-cancer patients were assessed for these enzymes. The mean activity of the antioxidant and depleting enzyme GSH-peroxidase was elevated significantly by twofold in the cancer tissue compared to normal tissue. However, the activity of the replenishing enzyme GSSG-reductase and the level of the depleting enzyme GSH-s-transferase P1-isoenzyme were significantly reduced by 23% and 33% respectively. As in a previous paper we found that GSH was depleted and γ-glutamine transpeptidase was diminished in oesophageal cancer. There can be two reasons for GSH depletion. Firstly, elevated GSH–peroxidase will use more GSH in an attempt to cope with the excessive production of oxyradicals as revealed by elevated lipid peroxidation; this was, as shown by us before, elevated sixfold in oesophageal cancer. Secondly, if little replenishment of GSH occurred the level of GSH would become lower. This was confirmed by our findings that the activities of the replenishing enzymes were significantly diminished in oesophageal cancer tissue. Contrary to what was expected, the other depleting enzyme GSH-s-transferase P1 was not elevated in cancer tissue but was significantly lower. However, in the blood of the same patients it was significantly elevated. An explanation for this phenomenon is that, although the production of GST-P1 was enhanced in cancer, it did not show because it was rapidly extruded into the blood by an unknown mechanism operational only in cancer cells.

**Keywords:** GSH-peroxidase; GSSG-reductase; GSH-s-transferase P1-isoenzyme; oesophageal cancer

Cancer in humans and animals is a multistage disease which results from multiple mutational events (Guyton and Kensler, 1993). Reactive oxygen species (ROS) (Wagner et al, 1992), lipid hydroperoxides (Ames et al, 1993) and malondialdehyde (MDA) (Mukai and Goldstein, 1975; Basu and Mannett, 1983) are mutagenic. Therefore they are mediators of phenotypic and genotypic alterations, due to multiple single mutations, which will eventually result in neoplasia events (Guyton and Kensler, 1993). Genes that are critically involved in cell growth are particularly vulnerable. Examples are the proto-oncogenes (Crawford et al, 1988) and the tumour suppressor genes (Ribeiro et al, 1996).

Lipid peroxidation (LPO) in cancer, because of uncontrolled free radical involvement, leads to several changes in cancer cells (Eriksson and Andersson, 1992). Glutathione (GSH) and GSH peroxidase (GS-Px) salvage the cell from LPO damage, particularly from damage to the membranes (Masotti et al, 1988). The salvaging process occurs when peroxy-polyunsaturated fatty acids (peroxy-PUFAs) are converted to hydroxy-FAs by GS-Px at the expense of GSH (Sevanian et al, 1983), rather than allowing LPO to go to completion with the production of short-chain FAs and MDA.

GSH and GS-Px also prevent other oxyradical damage, especially that inflicted on proteins and DNA. They acted as members of the antioxidant system of the cell by degrading hydrogen peroxide before it is converted to a hydroxyl radical (OH) (Halliwell and Gutteridge, 1992). Hydroxyl radicals can cause mutagenic damages to DNA by hydroxylation of the bases of DNA, especially guanine (G), to form 8-hydroxy-G (Floyd, 1990). This mutagenic event causes a change in base pairing from a G:C base pair to a 8-OH-G:A pair, which will eventually lead to a substitution of G by T in the subsequent progeny strand, causing a G to T transversion (Kasai and Nishimura, 1991).

The GSH/GSH-s-transferase (GST) system detoxifies mutagens, including those derived from ROS. In tumours, the GST P1-isoenzyme is strongly and persistently expressed (Campbell et al, 1991) but it was reported that the A- and M-isoenzymes appeared to be down-regulated or simply not expressed in many tumours (Gajewska and Szczypka, 1992). Miura et al (1997) suggested that the expression of GST-P1 may be linked directly to malignant transformations and may be regulated by oncogenes such as c-*jun* and c-*fos* (Tsuchida and Sato, 1992). In gastric cancer tissue the expression of GST-P1 was increased at the expense of GST-A (Peters et al, 1990). However, it was found that in lung cancer, renal cancer and hepatomas the expression of

*Received 14 October 1997*
*Revised 21 October 1998*
*Accepted 5 November 1998*

*Correspondence to:* MMJ Oosthuizen

GST-P1 was both increased and reduced (Gajewska and Szczypka, 1992). What is the case then in oesophageal cancer? In 80% of the patients, the resected primary oesophageal cancer showed that the RNA transcript levels of GST-P1 were significantly elevated (Ishioka et al, 1991). However, in contrast, Sasano et al (1993) found that with both in situ hybridization and immunohisto chemistry assessments, GST-P1 was detected as strongly in normal oesophageal mucosa as in cancer. Only 35% of cases showed an increase of GST-P1 in cancer compared to normal tissue. Peterset al (1993) also found that GST-P1 levels were low in a high number of patients with oesophageal cancer. Why then the apparent high level of GST-P1 expression but low amounts present in cancer tissue? The answer might be found in the observation that patients with oesophageal cancer have significantly higher levels of GST-P1 in serum or plasma, which revert back to a normal level after surgical removal of the tumour (Niitsu et al, 1989; Tsuchida et al, 1989; Fan et al, 1995). This elevated extracellular or plasma GST-P1 enzyme is not the active 46 kDa dimer enzyme but the inactive monomer of 23 kDa. This monomer is not released in plasma as a result of the disruption of cells but due to an energy-dependent efflux process in the cancer cells. In normal subjects the GST-P1 monomer originates from aggregating platelets and is responsible for a low background level of GST-P1 in plasma.

We previously found that cancerous tissue of the oesophagus became depleted of GSH (Hunter et al, 1992). The extracellular GSH replenishing enzyme gamma-glutamyl transpeptidase ($\gamma$-GT) was also significantly depleted (Hunter et al, 1992). The aim of the present study was to extend the investigation on the intracellular depletion and replenishment of GSH in oesophageal cancer. Therefore we assessed the activity of one other replenishing enzyme GSSG-reductase. The specific activity and levels of the GSH-depleting enzymes GS-Px, A-, M- and P-isoenzymes of GSH-s-transferase (Hayes and Pulford, 1995) were also determined respectively.

**Table 1** Characteristics of groups studied

| Patients | Cancer group | Control group |
|---|---|---|
| Numbers | 31 | 29 |
| Average age (mean ± SEM) | 54 ± 5 | 52 ± 8 |
| Male/female ratio | 21/10 | 18/11 |

### MATERIALS AND METHODS

### Clinical material

Biopsy specimens were obtained from Black patients who underwent endoscopy for dysphagia at the gastroenterology clinic at Baragwanath Hospital, Soweto, South Africa. Patients with alleged cancer of the oesophagus (cancer group) and volunteers

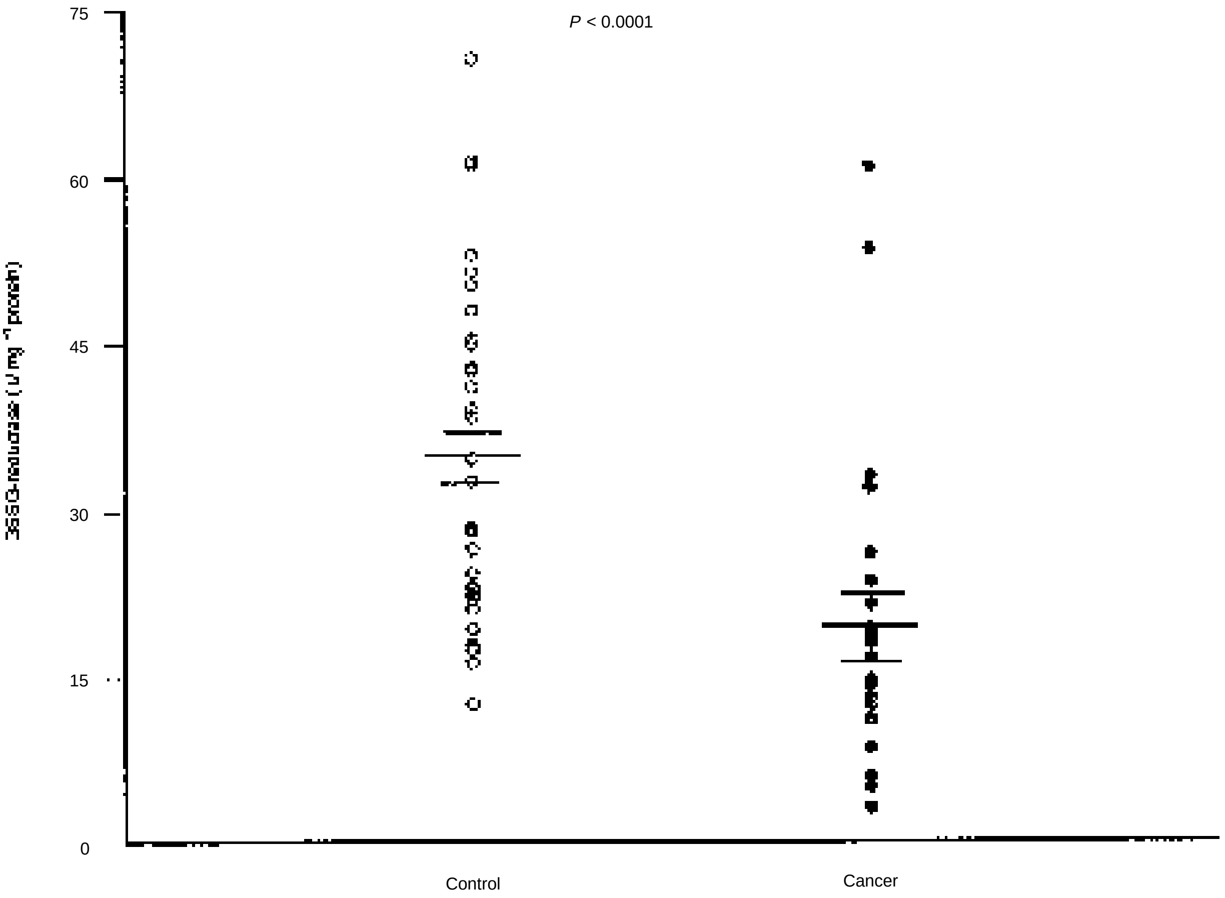

**Figure 1** GSSG-reductase activity in oesophageal tissue from normal and cancer patients. (≡) mean ± SEM

*© Cancer Research Campaign 1999*

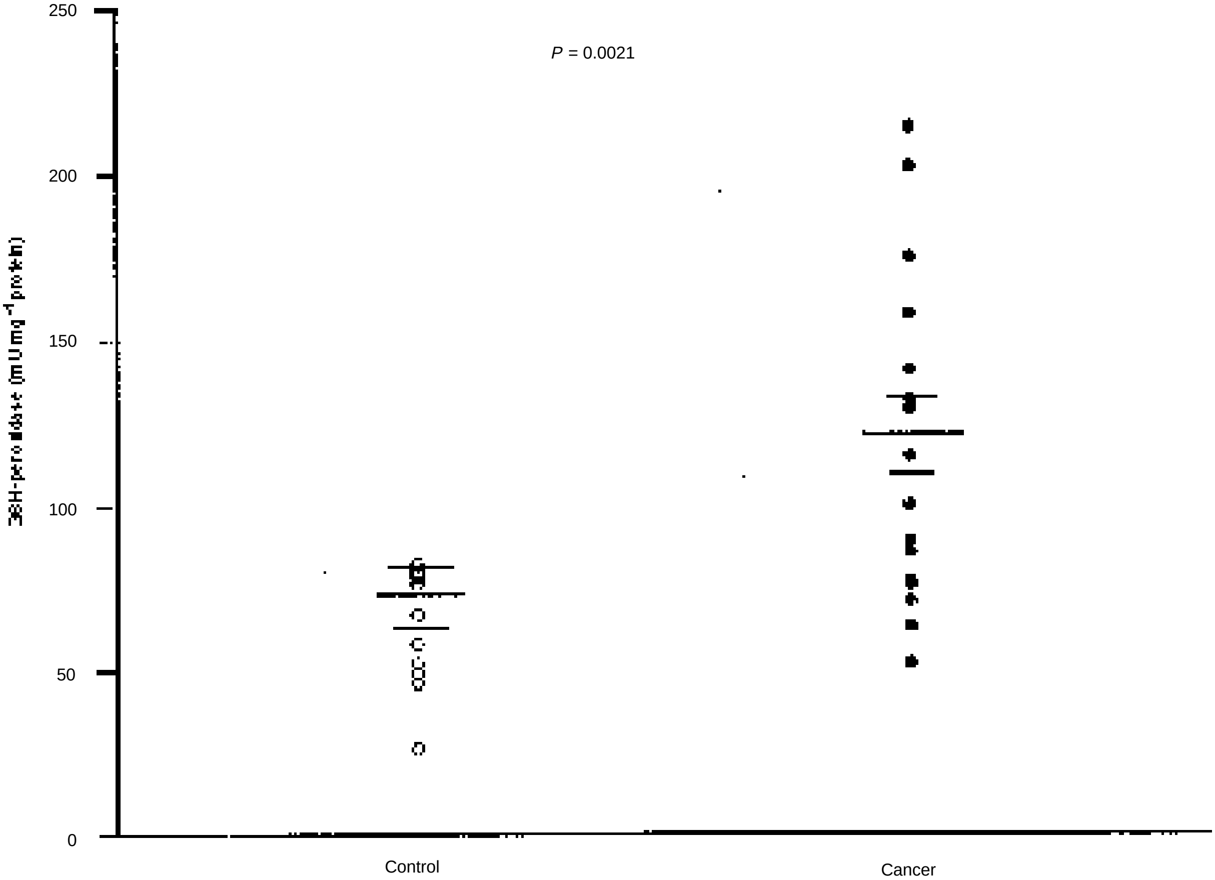

**Figure 2** GSH-peroxidase activity in oesophageal tissue from normal and cancer patients. (≡) mean ± SEM

having other gastroenterological symptoms but with normal oesophagogastro-duodenoscopy (control group) were subjected to biopsies. Normal tissue was not taken from the same cancer patient to serve as its own control. The reason for that was that the tumour could migrate upwards while that part of the oesophagus might still look normal macroscopically. Biopsies further down the oesophagus, beyond the cancer plaque were very difficult to obtain as most of the patients were obstructed. The total number of patients in the cancer group was 31 and in the control group was 29. The sex and mean age for the two groups were similar (see Table 1). Biopsy specimens were taken for histological and biochemical evaluation. The latter was performed only after it was confirmed that the cancer was squamous carcinoma. All patients with squamous carcinoma had advanced disease (stages 4 and 5; using the Japanese system). Human Ethics Committee approval was obtained from the University and Hospital, and informed consent obtained from all patients.

### Biochemical analysis

Immediately after tissue biopsies and blood were taken, the plasma and specimens for biochemical assessments were snap-frozen in liquid nitrogen and stored at –70°C until analysed. Tissue specimens were pulverized in a pre-cooled Besman tissue pulverizer at –80°C and extracted with cold buffered saline on a roller-mixer at 4°C for 30 min. After centrifugation at 9000 ***g*** for 15 min at 4°C the supernatant was analysed within 1 h for the GSH-linked enzymes at 37°C and its protein content.

The activity of GS-Px was determined by the method of Flohé and Gunzler (1984). With this method GSSG formation was continuously monitored in the presence of hydrogen peroxide, GSH, NADPH and added GS-R. The reduction of GSSG and subsequent oxidation of NADPH was monitored at $A_{340}$nm.

The activity of GS-R was determined by the method of Beutler (1969), which was essentially the same as for GS-Px except that GSSG was added and GS-R was omitted. FAD was also added to this assay to ensure that maximum GS-R activity was generated.

The levels of A-, M- and P-isoenzymes of GST were determined with a quantitative enzyme immunoassay (EIA) obtained from Biotrin International, Ireland which was used before by others (Brockmöller et al, 1993; Rees et al, 1995; Vaubourdolle et al, 1996; Vistisen et al, 1997). GSH and γ-GT were determined similarly as before by Hunter et al (1992) (results not included). Protein was determined by the Lowry et al method (1951).

### Statistical analysis

The mean of triplicate values for each specimen was subjected to the non-parametric test of Mann–Whitney to compare cancer and control groups. A *P*-value < 0.05 was accepted as statistically significant.

© Cancer Research Campaign 1999

**Table 2** Percentage elevated GSH-s-transferase P-isoenzyme content or expression in tissue and blood of patients with oesophageal and other cancers

| Cancer | Sample type | Number of patients | High GST-P mRNA (% of patients) | High GST-P protein content (% of patients) | Fold elevation | References |
|---|---|---|---|---|---|---|
| (a) Oesophageal | | | | | | |
| tissue | (i) | 31 | – | 27 | 1.3× | * |
| | (ii) | 43 | – | – | 6.0× | Tsuchida et al, 1989 |
| | (iii) | 25 | 80 | – | – | Ishioka et al, 1993 |
| | (iv) | – | – | 35 | – | Sasano et al, 1993 |
| Oesophageal | (i) | 31 | – | 77 | 2.2× | * |
| blood | (ii) | 15 | – | 53 | – | Niitsu et al, 1989 |
| | (iii) | 43 | – | 60 | – | Tsuchida et al, 1989 |
| | (iv) | 135 | – | 75 | – | Fan et al, 1995 |
| Bronchus | Plasma | 29 | – | 65 | – | Howie et al, 1990 |

*This paper.

### RESULTS AND DISCUSSION

The activity of one of the GSH-replenishing enzymes – glutathione-reductase – was, contrary to what was found by El-Sharabasy et al (1993) in breast cancer, significantly reduced in oesophageal cancer (Figure 1). In addition to this, the intracellular replenishment of GSH was hampered further because we found this time, as well as previously, that γ-GT, the enzyme responsible for GSH influx from extracellular sources, was again diminished in oesophageal cancer (Hunter et al, 1992).

GSH-peroxidase, one of the GSH-depleting enzymes, is a more efficient metabolizer of hydrogen peroxide than catalase (CAT) (Simmons and Jamall, 1988). GS-Px is more important than either superoxide dismutase (SOD) or CAT in preventing peroxidation. We have found that the activity of GS-Px was significantly increased in oesophageal cancer tissue compared to normal tissue (Figure 2). This was in contrast to what was found in colon, cervical, thyroid and laryngeal cancer where the cancer group had a lower GS-Px activity than the control group (Jendryczko et al, 1993; Mulder et al, 1995; Bhuvarahamurthy et al, 1996; Durak et al, 1996). The latter finding was supported by other researchers who found that the remote humoral effect of extra-hepatic cancers was that the activities of the liver pro-oxidant enzymes were increased and the activities of the antioxidant enzymes were decreased. For example, they found in tumour-bearing rats injected with hTNF-α that the SOD and GS-Px activities decreased while the activity of xanthine oxidase increased (Theologides et al, 1994). Selenium deficiency, as was found in Chinese cancer patients by other researchers (Chen et al, 1992; Wasowicz et al, 1994), did not play a roll in cancer of Africans since we found the activity of SeGS-Px was higher in oesophageal cancer tissues than in normal tissue. Therefore, we believe that the enhanced activity of GS-Px in oesophageal cancer tissue of Black patients was rather due to an attempt of the cancer to cope with the excessive production of oxyradicals. This excessiveness was substantiated by a six times greater lipid peroxidation in oesophageal cancer compared to normal tissue (Levy et al, 1998). To avert oxyradical damage to membrane lipids, all cells including cancer cells had to maintain a high GS-Px activity and GSH content to prevent irreversible lipid peroxidation of PUFAs which formed short-chain FAs and MDA eventually. However, we found, as before, that GSH is not properly replenished in oesophageal cancer (Hunter et al, 1992) and since both GS-Px and GST are dependent on GSH to perform their antioxidant and detoxification function they become non-functional as soon as GSH becomes depleted; thus rendering the cancer cells unprotected against oxyradicals.

The GSH-s-transferase A-, M- and P-isoenzymes were the other GSH-depleting enzymes that we investigated. Campbell et al (1991) found that the isoenzyme GST-P1 was expressed regularly compared to GST-A which was found only in some neoplasms and GST-M which was seldom present. This was confirmed by our results where we found that the levels of GST-P1 were high enough to be detected while those of the GST-A and M-isoenzymes were not, despite the low detection limit of 250 ng $l^{-1}$ for the two EIAs. However, we have found that the level of GST-P1 in cancerous tissue was significantly lower than the level in normal oesophageal tissue (Figure 3). This was contrary to what others have found (Tsuchida et al, 1989; Ishioka et al, 1991) although they determined blood and not tissue levels. However, our results were not unexpected since a phenomenon was observed in cancer, where GST-P1 was extruded into the blood as the inactive monomer (Kura et al, 1996). That would have left the cancer tissue depleted of the dimer despite a high expression of GST-P1 mRNA. This might be the reason a consistent high level of immuno-detectable GST-P1 was found in plasma and serum of patients with oesophageal and other cancers (Niitsu et al, 1989; Tsuchida et al, 1989; Howie et al, 1990; Fan et al, 1995) (Table 2). Our results, as obtained from plasma of oesophageal and control patients, support the concept that GST-P1 was enhanced in cancer but did not show because it was rapidly extruded into the blood by an unknown mechanism operational only in cancer cells (Table 3; compare also 2(a)(i) with 2(b)(i) in Table 2.)

In summary, the depletion of the tissue antioxidant GSH supports the notion that oxyradicals in cancer are not under proper metabolic protection. GS-Px dispose of the excess pro-oxidant hydrogen peroxide, but as soon as GSH becomes depleted the

**Table 3** Blood levels of the GSH-s-transferase P1-isoenzyme in oesophageal cancer and normal patients expressed as μg $L^{-1}$

| | Cancer | Control |
|---|---|---|
| Mean | 32.3 | 17.5 |
| SEM | 3.7 | 1.5 |
| P-value (Mann–Whitney) | 0.0016* | – |

**P* < 0.01 difference highly significant.

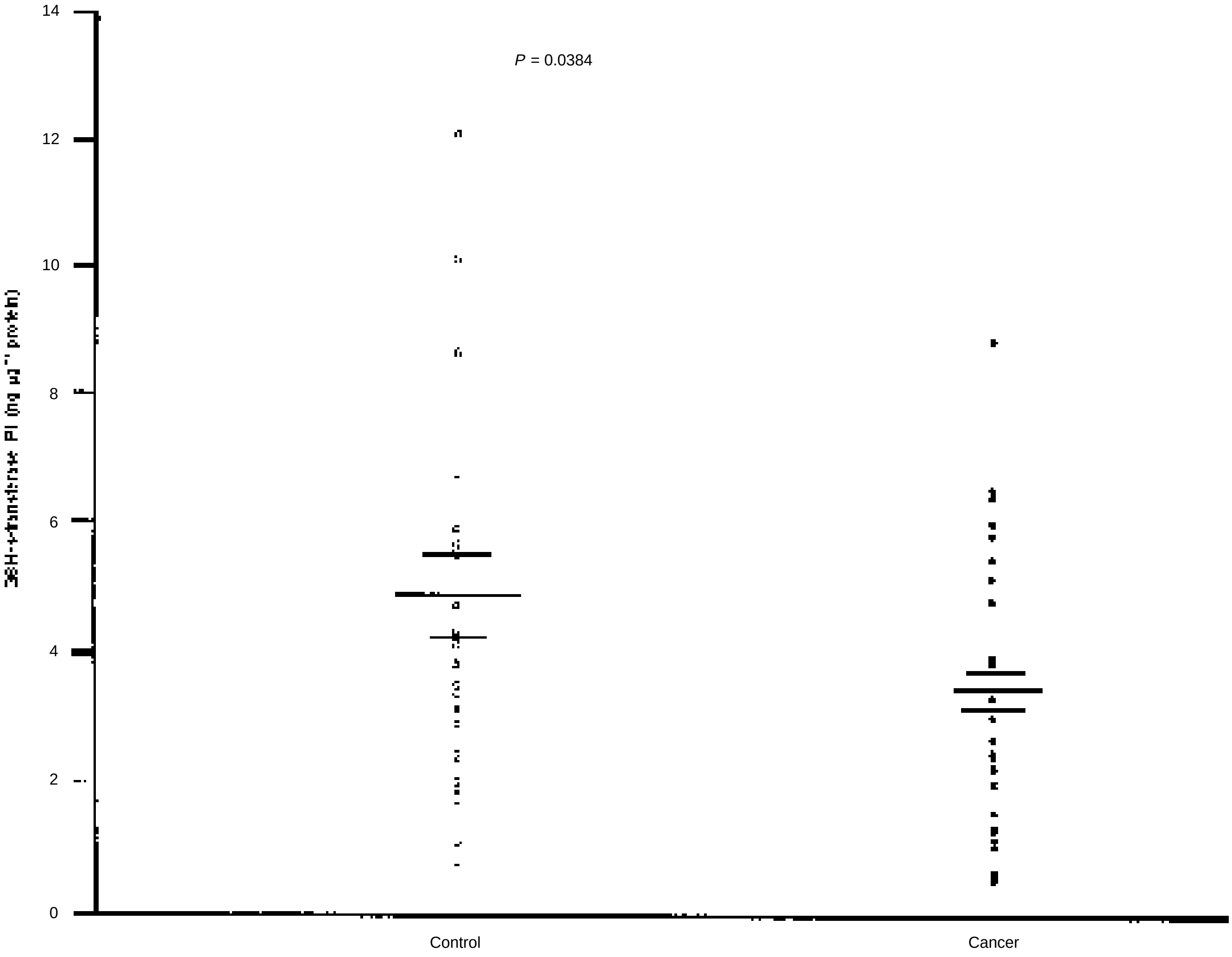

**Figure 3** GSH-s-transferase P1-isoenzyme levels in oesophageal tissue from normal and cancer patients. (≡) mean ± SEM

activities or levels of both GS-Px and GST-P are of no consequence. Therefore more mutagenic events will occur since mutagens and oxyradicals cannot be detoxified or scavenged by the impaired enzymes essential for an adequate antioxidant defense system.

### ACKNOWLEDGEMENTS

The Janet Sceales-Antrobus Cancer Research Trust; the National Cancer Association of South Africa, the South African Medical Research Council and the University of the Witwatersrand.

### REFERENCES

Ames BN, Shigenaga MK and Hagen TM (1993) Oxidants, antioxidants, and the degenerative disease of ageing. *Proc Natl Acad Sci USA* **90**: 7915–7922

Basu AK and Marnett LJ (1983) Unequivocal demonstration that malondialdehyde is a mutagen. *Carcinogenesis* **4**: 331–334

Beutler E (1969) Effect of flavin compounds on glutathione reductase activity: in vivo and in vitro studies. *J Clin Invest* **48**: 1957–1966

Bhuvarahamurthy V, Balasubramanian N and Govindasamy S (1996) Effect of radio- and chemotherapy on circulating antioxidant-system of human uterine cervical carcinoma. *Mol Cell Biochem* **158**: 17–23

Brockmöller J, Kerb R, Drakoulis N, Nitz M and Roots I (1993) Genotype and phenotype of glutathione s-transferase class μ isoenzymes μ and ψ in lung cancer patients and controls. *Cancer Res* **53**: 1004–1011

Campbell JAH, Corrigall AV, Guy A and Kirsch RE (1991) Immunohistologic localization of Alpha, Mu, and Pi class glutathione s-transferases in human tissues. *Cancer* **67**: 1608–1613

Chen J, Geissler C, Parpia B, Li J and Campbell TC (1992) Antioxidant status and cancer mortality in China. *Int J Epidemiol* **21**: 625–635

Crawford D, Amstad P, Zbindin I and Cerutti P (1988) Oxidant stress induces the proto-oncogenes c-*fos* and c-*myc* in mouse epidermal cells. *Oncogene* **3**: 27–32

Durak I, Bayram F, Kavutcu M, Canbolat O and Ozturk HS (1996) Impaired enzymatic antioxidant defense mechanism in cancerous human thyroid tissues. *J Endocrinol Invest* **19**: 312–315

El-Sharabasy MMH, El-Dosoky I, Horria H, and Khalaf AH (1993) Elevation of glutathione, glutathione-reductase and nucleic acids in both normal tissues and tumour of breast cancer patients. *Cancer Lett* **72**: 11–15

Eriksson LC and Andersson GN (1992) Membrane biochemistry and chemical hepatocarcinogenesis. *Crit Rev Biochem Mol Biol* **27**: 1–55

Fan KC, Huang YC and Li CH (1995) Radioimmunoassay for plasma glutathione S-transferase-pi and its clinical application in gastrointestinal cancer. *Cancer* **76**: 1363–1367

Flohé L and Günzler WA (1984) Assays of glutathione peroxidase. *Methods Enzymol* **105**: 114–121

Floyd RA (1990) The role of 8-hydroxyguanine in carcinogenesis. *Carcinogenesis* **11**: 1447–1450

© Cancer Research Campaign 1999

Gajewska J and Szczypka M (1992) Role of pi form of glutathione s-transferase (GST-pi) in cancer: a minireview. *Mater Med Pol* **24**: 45–49
Guyton KZ and Kensler TW (1993) Oxidative mechanisms in carcinogenesis. *Br Med Bull* **49**: 523–544
Halliwell B and Gutteridge JMC (1992) Biologically relevant metal ion-dependent hydroxyl radical generation. *FEBS Lett* **307**: 108–112
Hayes JD and Pulford DJ (1995) The glutathione s-transferase supergene family: regulation of GST and the contribution of the isoenzymes to cancer chemoprotection and drug resistance. *Crit Rev Biochem Mol Biol* **30**: 445–600
Howie AF, Douglas JG, Fergusson RJ and Beckett GJ (1990) Measurement of glutathione s-transferase Pi isoenzyme in plasma, a possible marker for adenocarcinoma of the lung. *Clin Chem* **36**: 453–456.
Hunter SJS, Richards GA, Oosthuizen MMJ and Bremner CG (1992) Glutathione, glutathione s-transferase and gamma-glutamyl transpeptidase levels in squamous cell carcinoma of the esophagus. *Res Surg* **4**: 160–164
Ishioka C, Kanamaru R, Shibata H, Konishi Y, Ishikawa A, Wakui A, Sato T and Nishihira T (1991) Expression of glutathione s-transferase-π messenger RNA in human esophageal cancers. *Cancer* **67**: 2560–2564
Jendryczko A, Pardela M and Kozlowski A (1993) Erythrocyte glutathione peroxidase in patients with colon cancer. *Neoplasma* **40**: 107–109
Kasai H and Nishimura S (1991) Formation of 8-hydroxydeoxyguanosine in DNA by oxygen radicals and its biological significance. In *Oxidative Stress: Oxidants and Antioxidants*, H Sies (Ed), pp. 99–116. Academic Press: London
Kura T, Takahashi Y, Takayama T, Ban N, Saito T, Kuga T and Niitsu Y (1996) Glutathione s-transferase-π is secreted as a monomer into human plasma by platelets and tumor cells. *Biochim Biophys Acta* **1292**: 317–323
Levy RD, Oosthuizen MMJ, Degiannis E and Lambrechts H (1998) Elevated reversible and irreversible lipid peroxidation in human oesophageal cancer. *Anticancer Res* **18**: 1325–1328
Lowry OH, Rosebrough NJ, Farr AL and Randall RJ (1951) Protein measurement with the Folin phenol reagent. *J Biol Chem* **193**: 265–275
Masotti L, Casali E, Gesmundo N, Sartor G, Galeotti T, Borrello S, Piretti MV and Paglinca G (1988) Lipid peroxidation in cancer cells: chemical and physical studies. *Ann N Y Acad Sci* **551**: 47–57
Miura K, Suzuki S, Tanita J, Shinkawa H, Satoh K and Tsuchida S (1997) Correlated expression of glutathione s-transferase-pi and c-Jun or other oncogene products in human squamous cell carcinomas of the head and neck: relevance to relapse after radiation therapy. *Jpn J Cancer Res* **88**: 143–151
Mukai FH and Goldstein BD (1975) Mutagenicity of malondialdehyde, a decomposition product of peroxidized polyunsaturated fatty acids. *Science* **191**: 868–869
Mulder TP, Manni JJ, Roelofs HM, Peters WH and Wiersma A (1995) Glutathione peroxidases in human head and neck cancer. *Acta Otolaryngol* **115**: 331–333
Niitsu Y, Takahashi Y, Saito T, Hirata Y, Arisato N, Maruyama H, Kohgo Y and Listowsky I (1989) Serum glutathione-s-transferase-π as a tumor marker for gastrointestinal malignancies. *Cancer* **63**: 317–323
Peters WHM, Wormskamp NGM and Thies E (1990) Expression of glutathione s-transferases in normal gastric mucosa and in gastric tumors. *Carcinogenesis* **11**: 1593–1596
Peters WHM, Wobbes T, Roelofs HMJ and Jansen JBMJ (1993) Glutathione s-transferases in esophageal cancer. *Carcinogenesis* **14**: 1377–1380
Rees GW, Trull AK and Doyle S (1995) Evaluation of an enzyme-immunometric assay for serum α-glutathione s-transferase. *Ann Clin Biochem* **32**: 575–583
Ribeiro U, Safatle-Ribeiro AV, Posner MC, Rosendale B, Bakker A, Swalsky PA, Kim R, Reynolds JC and Finkelstein SD (1996) Comparative p53 mutational analysis of multiple primary cancers of the upper aerodigestive tract. *Surgery* **120**: 45–53
Sasano H, Miuazaki S, Shiga K, Goukon Y, Nishihira T and Nagura H (1993) Glutathione s-transferase in human esophageal carcinoma. *Anticancer Res* **13**: 363–368
Sevanian A, Muakkassah-Kelly SF and Montrestrugue S (1983) The influence of phospholipase $A_2$ and glutathione peroxidase on the elimination of membrane lipid peroxides. *Arch Biochem Biophys*. **223**: 441–452
Simmons TW and Jamall IS (1988) Significance of alterations in hepatic antioxidant enzymes. *Biochem J* **251**: 913–917
Theologides A, Ingersoll-Stroubos AM and Apple FS (1994) TNF-alpha effect on oxygen free radical scavenging and generating enzymes in rat liver. *Biochem Mol Biol Int* **33**: 205–210
Tsuchida S and Sato K (1992) Glutathione transferases and cancer. *Crit Rev Biochem Mol Biol* **27**: 337–384
Tsuchida S, Sekine Y, Shineha R, Nishihira T and Sato K (1989) Elevation of the placental glutathione s-transferase from (GST-π) in tumour tissues and the levels in sera of patients with cancer. *Cancer Res* **49**: 5225–5229
Vaubourdolle M, Chazouillè V, Lasnier E, Serfaty I, Giboudeau J and Poupon R (1996) Plasma π-glutathione S-transferase as a marker of biliary cell damage. *Hepatology* **24**: 593A
Vistisen K, Priemé H, Okkels H, Vallentin S, Loft S, Olsen JH and Poulsen HE (1997) Genotype and phenotype of glutathione s-transferase-μ in testicular cancer patients. *Pharmacogenetics* **7**: 21–25
Wagner JR, Hu C-C and Ames BN (1992) Endogenous oxidative damage of deoxycytidine in DNA. *Proc Natl Acad Sci USA* **89**: 3380–3384
Wasowicz W, Gromadzinska J, Sklodowska M and Popadiuk S (1994) Selenium concentration and glutathione peroxidase activity in blood of children with cancer. *J Trace Elem Electrolytes Health Dis* **8**: 53–57

© Cancer Research Campaign 1999
